# Residue analysis and persistence evaluation of fipronil and its metabolites in cotton using high-performance liquid chromatography-tandem mass spectrometry

**DOI:** 10.1371/journal.pone.0173690

**Published:** 2017-03-14

**Authors:** Xiaohu Wu, Yang Yu, Jun Xu, Fengshou Dong, Xingang Liu, Pengqiang Du, Dongmei Wei, Yongquan Zheng

**Affiliations:** 1 Institute of Plant Protection, Chinese Academy of Agricultural Sciences, State Key Laboratory for Biolog of Plant Diseases and Insect Pests, Haidian District, Beijing, China; 2 Solid Waste and Chemicals Management Center, Ministry of Environmental Protection, Yuhuinanlu No.1, Chaoyang District, Beijing, Beijing, China; The University of Melbourne, AUSTRALIA

## Abstract

A simple residue analytical method based on the QuEChERS approach and high-performance liquid chromatography-tandem mass spectrometry (UPLC-MS/MS) detection was developed for the analysis of fipronil and its three metabolites in cottonseed, cotton plant and soil. The average recoveries of four test compounds from all three matrices were 78.6–108.9% at the level of 0.005 to 0.5 mg/kg, with an RSD in the range of 0.6 to 13.7%. The limit of quantification (LOQ) of the four test compounds ranged from 0.005 to 0.01 mg/kg. The results of the residual dynamics experiments showed that fipronil dissipated rapidly in cotton plants and soil and that oxidation and photolysis were the main degradation pathways. Moreover, the bi-exponential models demonstrated a good fit of the measured data for fipronil in cotton plants and soil, with R^2^ in the range of 0.8989 to 0.9989. Furthermore, a total of 40 samples of cottonseed from Shandong Province were analyzed, and all of the samples were free from the four test compound residues.

## Introduction

China, the world's largest cotton producer, generates more than a quarter of the global cotton output (FAOSTAT, 2014) [1]. The byproduct cottonseed hull and cottonseed are the most efficient substrate materials for oyster mushroom and cotton seed, respectively. During cotton cultivation, cotton seedlings grown in warm, dry regions are susceptible to many soil pests, such as white grubs, Adgrotis ypsilon (Rottemberg), and wireworm resulting in decreased yield. Seed chemical treatment controls soil pests in an economic and simple way. Fipronil, a member of the phenyl pyrazole class of pesticides, [(±)-5-amino-1-(2,6-dichloro-a,a,a-trifluoro-p-tolyl)-4-trifluoromethylsulfinylpyrazole-3-carbonitrile], is a GABA-gated chloride channel antagonist. Fipronil binds to some sites on γ-aminobutyric acid (GABA), blocking the passage of chloride ions, resulting in excessive release of neuronal stimulation and death of the target insect [2]. Fipronil has been effectively delivered to target pests via soil, foliar, bait or seed treatment and has been found to be effective against many species of soil insects [3]. Liu et al. [4] reported that 5% fipronil FSC at 1 g fipronil/1 kg seed controlled the peanut wireworm during full growing season, with control efficacy of 98.53% and yield increment of 33.33%.

However, fipronil exhibits severe toxicity and adverse effects to non-target organisms. Some studies indicate that exposure to fipronil can pose a risk for terrestrial game birds, honeybees, aquatic animals and marine invertebrates [5]. Moreover, fipronil is degraded by reduction, oxidation and photolysis to produce a variety of metabolites. The degradation products (metabolites) of fipronil are more toxic than the parent compound [6]. Desulfinyl (MB46513) was found to be a product of photolysis [7] and has a higher acute toxicity to mammals than fipronil itself by a factor of 10 (Pesticide Action Network-UK (PAN), 2000). Fipronil can undergo biological oxidation or reduction to produce the respective sulfone (MB46136) or sulfide (MB45950) [8–9]. Gunasekara et al. [5] reported that MB46136 is 3.3 times more toxic to *L*. *macrochirus* (bluegill sunfish) and metabolites MB46136 and MB45950 are more toxic to freshwater invertebrates than fipronil. Therefore, it is important to establish a reliable analytical method of fipronil and its metabolites to obtain more accurate data in the evaluation of environment risks and food safety. Numerous methods have been reported for the analysis of fipronil and its metabolites in differentmatrices. Kadar and Faucon [10] analyzed fipronil and its metabolites in pollen based on a three-step liquid−liquid partitioning and two cleanup steps, in combination with LC-ESI-MS/MS. Wang et al. [11] developed a liquid–solid extraction combined with purification on florisil columns for the determination of fipronil and its metabolites in soil, maize stem and maize grain by GC-ECD. Bhardwaj et al. [3] and Mandal and Singh [12] developed a method for the determination of fipronil and its metabolites in soil and cabbage (*Brassica oleracea var*. *capitata* L.) by gas liquid chromatography (GLC) and confirmed the analysis by mass spectrometry (GC-MS). However, this method consumed large quantities of solvent and time. The QuEChERS (quick, easy, cheap, effective, rugged and safe) method has been widely used for the extraction of a variety of complex matrices prior to analysis. Samples were extracted with acetonitrile, followed by cleanup with dispersive SPE and analysis by mass spectrometry (MS). QuEChERS extraction followed by mass spectrometry analysis (LC-MS/MS or GC-MS) has been successfully used for the determination of fipronil and its metabolites in vegetables and fruit, corn, sugarcane juice, jiggery, peanut, and okra [6, 13–16].

In China, the fipronil is only approved for use on upland crops as a seed treatment (Announcement No. 1157 of the Ministry of Agriculture of the People’s Republic of China, 2009). Fipronil has been registered as a seed-dressing agent on corn and peanut to control various soil pests (Pesticide Electronic Handbook 2016) [17]. In addition, fipronil is in the process of being registered in China for use against the soil pests of cotton. Only one paper reported that the analytical methods developed for cotton lint and seed involved extraction of analytes with larger volume of solvents, followed by the traditional liquid–liquid partitioning and column cleanup with a mixed adsorbent (neutral alumina: Florisil: activated charcoal, 1:2:0.5 w/w) for the final determination in GC-ECD [18]; however, this method did not involve the determination of fipronil metabolites. Therefore, it is of great significance to develop a simple and sensitive analytical method for the determination of fipronil and its metabolites in cottonseed. The proposed method can be easily adapted to the analysis of fipronil and its metabolites in cottonseed.

## Materials and methods

### Materials

Fipronil (99.7% purity) and three metabolites (fipronil sulfone (MB46136, 99.7% purity), fipronil sulfide (MB45950, 97.1% purity) and fipronil desulfinyl (MB46513, 97.8% purity) were purchased from Rhone-Poulenc Agro, Lyon, France. Acetonitrile (HPLC grade, ≥99.9%) was purchased from Sigma-Aldrich (Steinheim, Germany). Acetonitrile, MgSO_4_ and NaCl (analytical grade) were purchased from Beihua Fine-Chemicals Co. (Beijing, China). Ultra-pure water was purified using a MILLI-Q Pure treatment (Millipore, USA). PSA and GCB (40 μm) sorbents and 0.22-μm nylon syringe filters were purchased from Agela Technologies Inc. (Agela, Tianjin, PRC).

Standard stock solutions (100 mg/L) of fipronil and its metabolites were prepared in acetonitrile and were stored at -20°C. The working solutions were prepared by serial dilution of stock solution to 0.005, 0.01, 0.05, 0.1, 0.25, 0.5, 1 and 10 mg/L with acetonitrile and were stored at 4°C before use.

### Sampling

The cotton plant and soil samples were collected from our residual trial field. A supervised field trial was performed during 2014−2015 in Shandong (N36°41′, E118°55′) and Henan Provinces (N35°02′, E112°44′) according to “Standard operating procedures on pesticide registration residue field trials” issued by the Institute of the Control of Agrochemicals, Ministry and Agriculture, The People’s Republic of China. Our study was carried out on private land, and the owner of the land gave permission to conduct the study on this site. To investigate the dissipation curves of fipronil and its three metabolites in cotton plants, cottonseeds were coated with 20% fipronil flowable seed coating agent (provided by Sinochem Ningbo Chemicals Co., Ltd) at a dosage of 7.5 g a.i. fipronil/100 kg seed (1.5 times the recommended dosage) before sowing. When the mean height was 10–15 cm, cotton plant samples (approximately 1000 g) were collected at random from each plot at time intervals of 2h, 1, 2, 4, 7, 14, 21 and 28 days. Each treatment field had three replicate plots, and each plot was separated by irrigation channels with an area of 30 m^2^. 90 g seeds were sown in each plot. In addition, to investigate the dissipation of fipronil in soil, 33.75 mg of the 20% fipronil flowable seed coating agent was sprayed on each soil plot, which correspond to 1.5 times the recommended dosage (7.5 g a.i. fipronil/100 kg seed). Approximately 1000g soil samples from a depth of 0-10cm were collected randomly from five points in each plot at 2h, 1, 2, 4, 7, 14, 21 and 28 days after soil spraying.

A total of 40 cottonseed samples were collected from different wet markets of Shandong Province during the year 2015. The cities involved were Qingdao, Weifang, Heze, Jining and Dongying. The sample size from each wet market was at least 500 g. After collection, the cottonseed samples were placed in an ice box and were transferred to the laboratory. Then, the cottonseed samples were stored at -20°C until analysis.

### Sample preparation, extraction and purification

The soil samples and cotton plants were collected from the field experiments, and cottonseed was collected from market. After collection, the soil samples were air-dried, crushed and sieved to pass through a 2 mm sieve to remove any plant tissue and stones. The cottonseed and cotton plant samples were chopped by high-speed homogenization. These samples were stored at −20°C before being analyzed.

Soil: A 10.0 g soil samplewas weighed into a 50 mL centrifuge tube, and 2 mL of distilled water was added, followed by the addition of 10mL acetonitrile. The centrifuge tubes were shaken at 25°C by tissue lyser (Thmorgan CK2000, China) for 10 min. After adding 2.0 g of NaCl and 4.0g of anhydrous MgSO_4_, the mixture was vortexed for 1 min and centrifuged at an RCF of 2,077 g for 5 min at 4°C. Then, 1.5 mL of the acetonitrile layer was transferred into a 2.0 mL dispersive-SPE tube containing 30 mg PSA and 150 mg MgSO_4_. The dispersive-SPE tube was shaken in a vortex for 1min and then centrifuged for 5min at 2,077 g. The supernatant solution was filtered through a 0.22μm polypropylene filter and transferred into an auto sampler vial for UPLC-MS/MS analysis.

Cottonseed and cotton plant: 5.0 g of homogenized sample (cottonseed and cotton plant) was put into a 50 mL centrifuge tube. Distilled water (5 mL for cottonseed and 10 mL for cotton plant) and acetonitrile (10 mL) were added. The rest of the procedure was the as for the soil sample.

### UPLC-MS/MS detection

All analysis was performed on a Waters Acquity UPLC system coupled to a triple-quadrupole Xevo-TQD equipped with an electrospray ionization source (ESI) (Waters Corp., Milford, MA, USA). Fipronil and its metabolites were separated on an Acquity UPLC BEH Shield RP18 column (50 × 2.1 mm, 1.7 μm particle size, Milford, MA, USA) with a gradient of acetonitrile (A)/water (B) at a flow rate of 0.3 mL min^−1^. The gradient elution program was as follows: 0–1.0 min 30–70% A; 1.0–3.5 min 70–95% A; 3.6–5.0 min 70% A. The injection volume was 7 μL, and the column oven was at 40°C.

The mass spectrometer was operated in ESI^-^ with multiple reactions monitoring (MRM) scan mode. The source temperature was set at 150°C, capillary voltage at 3.0 kV, desolvation temperature at 400°C, desolvation gas flow at 1000 L/h and cone gas flow at 50 L/h. Quantification was performed using ESI^−^ MRM mode with *m/z* 434.91→330.01 for fipronil, *m/z* 450.97→414.97 for MB46136, *m/z* 418.98→382.99 for MB45950 and *m/z* 387→351.01 for MB46513. The optimized cone voltages of 34, 36, 36 and 34 V for fipronil, MB46136, MB45950 and MB46513, and collision energies of 16, 26, 10 and 14 V were used for the four test compounds. The acquisition and analysis of data were performed with MassLynx version 4.1 software.

### Data analyses

The matrix effect (ME) was determined as follows:

$$ME\;(\%)=[\frac{slope\;in\;matrix}{slope\;in\;solvent}-1]\times 100$$ (1)

where ME (%) <0 indicates signal suppression and values >0 indicate ionization enhancement.

The first-order kinetics models and bi-exponential models were conducted to analyze the dissipation curves of fipronil. The first-order kinetics were analyzed using Microsoft Excel software and were represented as follows:

$$C={C_{0}e}^{-kt}$$ (2)

where C is the pesticide concentration (mg kg^−1^) at time t (d) after application, C_o_ is the initial concentration (mg kg^−1^), and k is the first-order rate constant (d^−1^).

The analysis of the bi-exponential kinetics was performed in KinGUIIv2.1 (BASF Corporation) and as follows:

$${C=A*e}^{-k1*t}+B*e^{-k2*t}$$ (3)

where C is the concentration at time t (d), A and B are constants, and k_1_ and k_2_ are the first-order rate and the second-order rate constant.

## Results and discussion

### Optimization of the LC-MS/MS conditions

**Fig 1:**
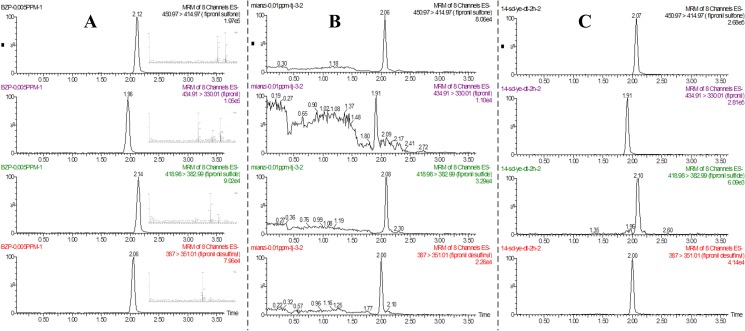
UPLC-MS/MS MRM chromatograms of fipronil and three metabolites of (A) standard (5 mg/kg), (B) cottonseed spiked at 10 mg/kg, (C) total ion chromatogram of the four test compounds in the 0-day (2 h) cotton plant sample.

GC combined with ECD, MS detectors [13, 18, 19] and LC-MS/MS [6, 20] presented a good response for fipronil and its metabolites in various matrices. However, UPLC-MS/MS provided higher sensitivity and selectivity of detection and shorter chromatography run-times than conventional GC or HPLC-MS techniques. In our study, the UPLC-ESI-MS mass spectra for the four compounds were investigated by direct injection at different ESI cone voltages, and higher sensitivity in the ESI^−^ mode was observed. Consequently, deprotonated molecular ions of the formula [M−H]^−^ were selected as precursor ions in MS–MS for the four compounds. Then, the precursor ions [M−H]^−^ were fragmented by collision-induced dissociation (CID), and the SRM was optimized to achieve the highest degree of sensitivity. The optimized MS/MS transitions and other optimal conditions for the analysis of fipronil and its three metabolites are summarized in Table 1. For fipronil and its metabolites, chromatographic separations were achieved on an Acquity UPLC BEH Shield RP18 column using a mobile phase of water and acetonitrile. As seen from Fig 1A, the chromatographic separation of four pesticides was completed in 2.5 min.

**Table 1:** UPLC-MS/MS conditions of fipronil and its three metabolites.

Compound	Molecular formula	Molecular weight	t_R_ (min)	Ion source	CV (V)	Quantification ion transition	CE1 (eV)	Confirmatory ion transition	CE2 (eV)
Fipronil	C_12_H_4_Cl_2_F_6_N_4_OS	435.9	1.92	ESI^-^	34	434.91 → 330.01	16	434.91 → 250.03	22
MB46136	C_12_H_4_Cl_2_F_6_N_4_O_2_S	451.93	2.06	ESI^-^	36	450.97 →414.97	26	450.97 → 282.03	26
MB45950	C_12_H_4_ Cl_2_F_6_N_4_S	419.94	1.98	ESI^-^	36	418.98 → 382.99	10	418.98→ 262	26
MB46513	C_12_H_4_ Cl_2_F_6_N_4_	387.97	1.88	ESI^-^	34	387→ 351.01	14	387→382.09	28

### Method validation

The linearity was determined using the peak areas of the product ion obtained from the MRM mode scan, and these results are summarized in Table 2. Matrix-matched standard calibration curves, consisting of seven concentration levels (0.005, 0.01, 0.05, 0.1, 0.25, 0.5 and 1 mg/L), were set up by plotting the analyte concentrations against peak areas, and the MS detector responses were linear from 0.005–0.25 mg/L (R^2^ = 0.9822–0.9981). However, matrix effects are one of the major drawbacks of LC-MS-MS, especially when working in ESI mode [21], and it may result in decreased or increased analyte signals, leading sometimes to erroneous results [22]. Moreover, matrix effect also strongly depends on the chemical nature of the pesticide, each matrix and sample treatment procedure, etc [23]. In consequence, the matrix effects (ME) were investigated for all samples by comparison of the slopes of the matrix-matched and solvent-based calibration curves. The results showed a remarkable %ME for fipronil and its three metabolites in all matrices in a range from -97.3% to +24.5%. Obvious decreases of signal intensity of the four test compounds were observed in cottonseed and cotton plant matrices (-95.3% ≤ME≤ -97.3% for cottonseed and -21.5% ≤ME≤ -81.1% for cotton plant). For soil matrices, MB46136 and MB45950 showed a decreased presence (-6.3%≤ME≤-39.1%), whereas fipronil and MB46513 showed a signal enhancement effect (+17.9%≤ME≤+24.5%). Therefore, matrix-matched calibration curves were used for accurate quantitation for fipronil and its three metabolites.

**Table 2:** Linear regression parameters of the calibration curve of fipronil and its three metabolites in all matrix matrices and solvents for 0.005–0.25 mg/kg.

Compound	Matrix	Regression equation	R^2^	Calibration range	LOQ (mg/kg)	Matrix effect
Fipronil	Solvent	y = 803022 x + 5090.1	0.9972	0.005–0.25	0.005	-
	cottonseed	y = 24376 x + 164.39	0.9981	0.005–0.25	0.01	-97.0
	soil	y = 1E+06 x—5963.3	0.9883	0.005–0.25	0.005	+24.5
	Cotton plant	y = 630068 x—5845.3	0.9822	0.005–0.25	0.01	-21.5
MB46136	Solvent	y = 969450 x + 10994	0.9904	0.005–0.25	0.005	-
	cottonseed	y = 26550 x + 163.95	0.9956	0.005–0.25	0.01	-97.3
	soil	y = 590805 x—2494.6	0.9911	0.005–0.25	0.005	-39.1
	Cotton plant	y = 536025 x—3565.1	0.9883	0.005–0.25	0.01	-44.7
MB45950	Solvent	y = 484062 x + 7400.4	0.9839	0.005–0.25	0.005	-
	cottonseed	y = 19054 x + 305.93	0.991	0.005–0.25	0.01	-96.1
	soil	y = 453503 x + 3846.2	0.9981	0.005–0.25	0.005	-6.3
	Cotton plant	y = 134565 x + 1975.8	0.9887	0.005–0.25	0.01	-72.2
MB46513	Solvent	y = 630425 x + 4177.5	0.9962	0.005–0.25	0.005	-
	cottonseed	y = 29858 x + 440.59	0.9917	0.005–0.25	0.01	-95.3
	soil	y = 743522 x—683.44	0.9966	0.005–0.25	0.005	+17.9
	Cotton plant	y = 119410 x + 1404.1	0.9911	0.005–0.25	0.01	-81.1

A recovery experiment was conducted to determine the accuracy and precision of the analytical method. At fortification levels of 0.01, 0.05 and 0.5 mg/kg in cottonseed and cotton plant, the average recoveries of fipronil and its three metabolites ranged from 78.6% to 108.5%, with relative standard deviations (RSD) of 1.9–13.1% (n = 5). The average recoveries of the four pesticides in soil at three fortified levels (0.005, 0.05, and 0.5 mg/kg) were 79.7–108.9%, with RSDs of 0.6–13.7% (n = 5) in soil (Table 3), which satisfied the requirements of the DG SANCO/12495/2011 guidelines (mean recovery between 70 and 120% and RSD≤20%). The limit of quantification (LOQ) was defined according to the SANCO Guidelines, where the lowest spiked levels gave satisfactory recoveries 70–120% and RSD≤20%; therefore, the LOQ of the method was 0.01 mg/kg in cottonseed and cotton plant and 0.005 mg/kg in soil. The results indicated that this method provides satisfactory precision and accuracy and can be used for the determination of fipronil and its three metabolites in cottonseed, cotton plant and soil.

**Table 3:** Recoveries (n = 5, percent) and relative standard deviation (±RSD) for fipronil and its three metabolites from three matrices in different spiked levels.

Matrix	Spiked level (mg/kg)	Mean Recovery (%) ± RSD (%)			
		Fipronil	MB46136	MB45950	MB46513
cottonseed	0.01	82.9±9.7	81.8±5.6	78.6±6.0	85.4±9.4
	0.05	82.9±10.1	93.6±9.5	91.4±8.5	84.5±11.0
	0.5	90.7±12.7	79.3±10.4	100.6±10.5	92.2±13.1
cotton plant	0.01	103.3±3.8	108.5±5.2	96.4±10.1	96.3±10.4
	0.05	99.8±5.1	100.3±4.5	92.8±1.9	87.4±3.7
	0.5	102.0±3.4	97.0±3.1	100.9±6.0	105.8±9.4
soil	0.005	95.8±10.4	94.5±8.8	108.9±10.5	105.6±13.7
	0.05	98.0±5.3	103.5±7.6	92.0±8.1	91.8±5.0
	0.5	91.7±0.6	98.5±2.1	92.6±3.8	79.7±3.5

### Persistence of fipronil and its metabolites in cotton plant and soil

**Fig 2:**
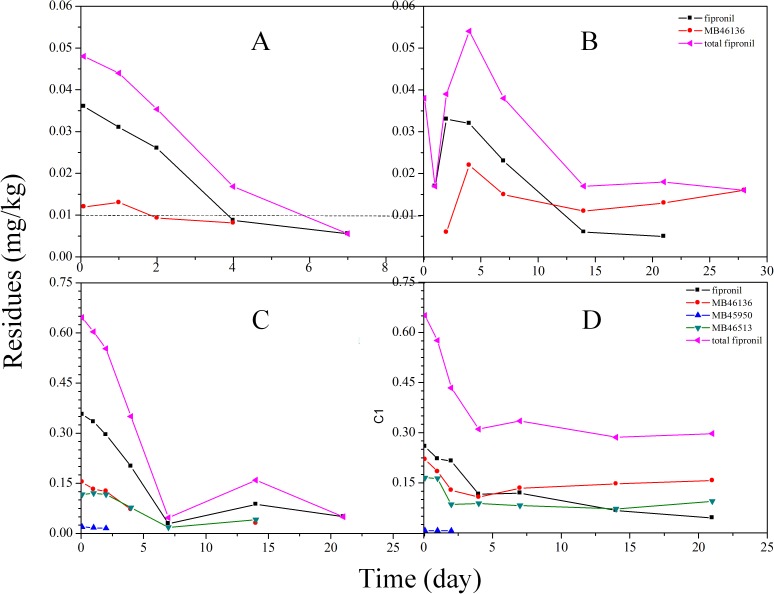
Dissipation of fipronil and three metabolites in cotton plant (A) and soil (B) at the Shandong site, 2014, and dissipation of the four test compounds in cotton plant (C) and soil (D) in Shandong, 2015.

**Fig 3:**
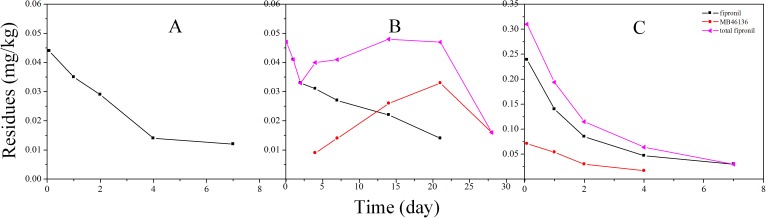
Dissipation of fipronil and three metabolites in cotton plant (A) and soil (B) at the Henan site, 2014, and dissipation of the four test compounds in cotton plant (C) in Henan, 2015.

The overall results of the analysis of cotton plant and soil after application of fipronil at 7.5 g a.i. fipronil/100 kg seed are presented in Fig 2 and Fig 3, and the half-lives and other statistical parameters were calculated and are summarized in Table 4. In 2014, the initial concentrations of total fipronil (sum of fipronil and its metabolites) on cotton plants were 0.048 mg/kg and 0.044 mg/kg at the Shandong and Henan sites, respectively. The residues of fipronil were mainly present as parent compounds along with a small amount of the metabolites, such as MB46136. Three other metabolites were also detected but remained at very low concentrations (<0.005 mg/kg) during the entire dissipation process. Moreover, the residues of fipronil dropped quickly and were <0.01 mg/kg after 7 days at both the Shandong and Henan sites. A similar trend of degradation was found in fipronil-treated soils (both Shandong and Henan Province). After 2 h of application, only the parent compound was detected. Metabolites MB46136 at the Shandong site were minimal 2 days after application, reached 0.022 mg/kg on day 4, and decreased over time, whereas at the Henan site, the corresponding values were 0.009 mg kg^−1^ on day 4 and increased until day 21 (0.033 mg/kg).

**Table 4:** Half-life (T_1/2_) and other statistical parameters for fipronil dissipation in the cotton field conditions.

Size	Year	Matrix	Compound	first-order kinetics equations			bi-exponential models		
				Regression equation	R^2^	T_1/2_	Regression equation	R^2^	T_1/2_
Shan dong	2014	Cotton Plants	fipronil	-	-	-	-	-	-
			Total	-	-	-	-	-	-
		Soil	fipronil	y = 0.034e^-0.096x^	0.8149	7.2	y = 0.034e^-0.096x^	0.8149	7.2
			Total	-	-	-	-	-	-
	2015	Cotton Plants	fipronil	y = 0.2652e^-0.095x^	0.536	7.3	y = 0.3492e^-0.2387t^ + 0.04329e^-2.22*10-14t^	0.9109	3.5
			Total	y = 0.5091e^-0.117x^	0.6289	5.9	y = 3.0788*10^-14^e^-0.6532t^ + 0.7018e^-0.1871t^	0.9047	3.7
		Soil	Fipronil	y = 0.2244e^-0.082x^	0.9345	8.5	y = 0.146e^-0.2987t^ + 0.1214e^-0.04569t^	0.9608	4.7
			Total	y = 0.4926e^-0.032x^	0.5651	21.7	y = 0.3910e^-0.4765t^+ 0.2926e^-2.221*10-14t^	0.9653	4.4
Henan	2014	Cotton Plants	fipronil	y = 0.0412 e^-0.199x^	0.9146	3.5	y = 0.03873e^-0.3352t^ + 0.007e^-2.22*10-14t^	0.9776	2.7
			Total	y = 0.0459e^-0.215x^	0.8851	3.2	y = 0.04707e^-0.2563t^ + 0.0019e^-2.22*10-14t^	0.8989	2.9
		Soil	fipronil	y = 0.0403e^-0.045x^	0.9212	15.4	y = 0.0127e^-0.903t^ + 0.03562e^-0.04t^	0.9848	3.9
			Total	-	-	-	-	-	-
	2015	Cotton Plants	fipronil	y = 0.1882e^-0.289x^	0.9261	2.4	y = 0.1991e^-0.7879t^ + 0.05304^e-0.0844t^	0.9999	1.2
			Total	y = 0.2672e^-0.329x^	0.9728	2.1	y = -0.2962e^-0.4239t^ +1.2962e^-0.2830t^	0.9989	1.3
		Soil	fipronil	-	-	-	-	-	-
			Total	-	-	-	-	-	-

In 2015, the residues of total fipronil observed on 0-day cotton plants were 0.646 mg/kg at the Shandong site. Fipronil residues were degraded to MB46136, MB45950 and MB46513. The amount of metabolite MB46136 was 0.154 mg/kg, followed by MB46513 at 0.117 mg/kg and MB45950 at 0.019 mg/kg. In contrast, at the Henan site, MB46136 was the predominant metabolite of fipronil and was found at a concentration of 0.071 mg/kg on 0-day (2 h) cotton plants. The parent compound and its metabolites decreased with time. After 28 d of application, fipronil and metabolites had levels lower than the LOQ. We observed that the rate of dissipation of the parent compound fipronil dissipated slower than metabolites MB46136 and MB46513 at the Shandong site. In contrast, a higher rate of dissipation of fipronil was observed at the Henan site. Furthermore, higher amounts of metabolites MB46136 and MB46513 were found after 0-day (2 h) of its application in soil of the Shandong site. Moreover, the presence of MB46136 in higher amount compared to MB46513 was found during the entire dissipation process. At the end of the experiment, fipronil and its metabolites were present in levels lower than the LOQ. However, no residues were found for the treatment in the soils at the Henan site during the whole period.

Fipronil degrades rapidly by means of reduction, hydrolysis, oxidation, and photolysis to form metabolites. Fipronil degradation is influenced by many factors, for example, sunlight, temperature, humidity, microorganisms, the pH of the soil and water, and the nature of the plant species [5]. Therefore, the difference in the degradation of fipronil in the cotton plant and soils of the Shandong and Henan sites may be due to different climatic conditions in Shandong and Henan Provinces. Moreover, higher amounts of fipronil sulfone were found in the cotton plants and soils, suggesting that oxidation-reduction played a major role in the metabolism of fipronil. In addition, fipronil desulfinyl was also produced, indicating that sunlight photolysis contributed to the dissipation of fipronil. Therefore, oxidation is a significant degradation pathway of fipronil, whereas photolysis is the second most important factor under different climate conditions in China. Similar results were observed in other experiments. Dutta et al. [24] observed that fipronil was degraded to sulfone and desulfinyl in cabbage, and the amount of fipronil sulfone residue was greater than desulfinyl. However, Bhardwaj et al. [3] and Li et al. [6] studied the dissipation of fipronil on cabbage and peanut seedlings, respectively, and found that desulfinyl was the main metabolite, followed by sulfone.

In general, the higher the R^2^ (0<R^2^<1), the better the model fits the experimental data. Considering the first-order kinetics models, at four experimental sites, the half-life of fipronil alone in cotton plants ranged from 2.4 to 7.3 days with an R^2^ 0.536–0.9261, and in soil, the half-life ranged from 7.2 to 15.4 days with an R^2^ 0.8149–0.9345. However, based on the bi-exponential model analysis, the half-life (T_1/2_) of fipronil was 1.2–3.5 d with an R^2^ of 0.9109–0.9999 in cotton plants and 3.9–7.2 d with an R^2^ of 0.8149–0.9848 in soil. For total fipronil, the correlation coefficient values in the four experimental sites, calculated using bi-exponential models, were also satisfactory (R^2^ = 0.8989–0.9989), and the observed half-life (T_1/2_) values were 1.3–3.7 days in cotton plants and 4.4 days in the soil of Shandong. Moreover, the half-life (T_1/2_) values obtained from the bi-exponential models were lower than the values obtained by the first-order models. A similar pattern of degradation was found for other pesticides [25–26]. Therefore, when the degradation deviates from first-order kinetic, the bi-exponential model should be taken into account to assess the kinetics of pesticide degradation.

### Monitoring of cottonseed samples

The analysis methods can be applied as a tool for monitoring the residues of fipronil and its metabolites in cottonseed samples. Among the 40 samples analyzed, the contents of the four compounds were all lower than the LOQ values.

## Conclusions

In this paper, a sensitive, simple method for the analysis of fipronil and its three metabolites in cottonseed, cotton plant and soil was developed, validated, and applied to analyze residues in field-incurred samples and market samples. The linearity, recoveries and LOQs were satisfactory for all of the matrices tested. At fourth experimental sites, the dissipation of fipronil in cotton plants and soil followed the bi-exponential kinetics with the half-life values varying from 1.2 to 3.5 days and 3.9 to 4.7 days, respectively. All of the cottonseeds collected from the wet markets were free from the four test compound residues. This method was simple and fast and should be used for checking the presence of traces of and its metabolites in cotton crops.

## Supporting information

S1 Table The data of the calibration curve for fipronil and its three metabolites in solvent for 0.005–0.25 mg/kg. (DOCX)

S2 Table The data of recoveries for fipronil and its three metabolites from three matrices in different spiked levels. (DOCX)

S3 Table The data of dissipation of fipronil and three metabolites in cotton plant and soil at the Shandong and Henan site. (DOCX)

S4 Table The data of residue residues of fipronil and its metabolites in cottonseed samples. (DOCX)
