# Supplementary material for: Residue analysis and persistence evaluation of fipronil and its metabolites in cotton using high-performance liquid chromatography-tandem mass spectrometry
Source: PLoS One. 2017 Mar 14;12(3):e0173690. doi: 10.1371/journal.pone.0173690 (PMC5349471; doi:10.1371/journal.pone.0173690)
Supplement: S1 Table — (DOCX) [file pone.0173690.s001.docx]

|  | fipronil | | MB46136 | | MB45950 | | MB46513 | |
| --- | --- | --- | --- | --- | --- | --- | --- | --- |
|  | Retention Time | Area | Retention Time | Area | Retention Time | Area | Retention Time | Area |
| BZP-0,005ppm-1 | 1.96 | 6087.712 | 2.12 | 8563.726 | 2.12 | 4463.333 | 2.03 | 4367.888 |
| BZP-0,005 ppm -2 | 1.94 | 5749.191 | 2.09 | 6508.687 | 2.14 | 4654.555 | 2.05 | 4335.17 |
| BZP-0,005 ppm -3 | 1.96 | 5677.273 | 2.12 | 7917.442 | 2.14 | 4444.242 | 2.04 | 4166.964 |
| BZP-0,01 ppm -1 | 1.96 | 9919.91 | 2.11 | 14709.88 | 2.14 | 8229.375 | 2.05 | 7901.155 |
| BZP-0,01 ppm -2 | 1.95 | 10913.97 | 2.11 | 15560.79 | 2.14 | 8521.194 | 2.04 | 8165.574 |
| BZP-0,01 ppm -3 | 1.94 | 10605.38 | 2.11 | 14774.04 | 2.13 | 8418.348 | 2.05 | 7993.917 |
| BZP-0,05 ppm -1 | 1.95 | 47671.88 | 2.11 | 66796.97 | 2.14 | 36460.91 | 2.04 | 39043.59 |
| BZP-0,05 ppm -2 | 1.95 | 47243.03 | 2.11 | 65528.8 | 2.14 | 35655.75 | 2.04 | 37130.04 |
| BZP-0,05 ppm -3 | 1.95 | 47590.18 | 2.11 | 65843.91 | 2.14 | 36631.3 | 2.04 | 37917.39 |
| BZP-0,1 ppm -1 | 1.94 | 91440.54 | 2.11 | 120569.6 | 2.14 | 62955.81 | 2.04 | 70978.23 |
| BZP-0,1 ppm -2 | 1.95 | 91151.19 | 2.11 | 121270.7 | 2.14 | 66036.41 | 2.04 | 73848.45 |
| BZP-0,1 ppm -3 | 1.95 | 93227.69 | 2.11 | 122918.1 | 2.14 | 64300.71 | 2.04 | 74216.82 |
| BZP-0,25 ppm -1 | 1.94 | 202450.3 | 2.11 | 249930.4 | 2.14 | 127712.4 | 2.04 | 159973.2 |
| BZP-0,25 ppm -2 | 1.94 | 197559.9 | 2.11 | 246346.1 | 2.14 | 123866.2 | 2.04 | 152024.4 |
| BZP-0,25 ppm -3 | 1.94 | 208826.4 | 2.11 | 244632.7 | 2.14 | 121312.4 | 2.04 | 165479.4 |

S1 Table The data of the calibration curve for fipronil and its three metabolites in solvent for 0.005-0.25 mg/kg
