# Supplementary material for: Residue analysis and persistence evaluation of fipronil and its metabolites in cotton using high-performance liquid chromatography-tandem mass spectrometry
Source: PLoS One. 2017 Mar 14;12(3):e0173690. doi: 10.1371/journal.pone.0173690 (PMC5349471; doi:10.1371/journal.pone.0173690)
Supplement: S2 Table — (DOCX) [file pone.0173690.s002.docx]

S2 Table The data of recoveries for fipronil and its three metabolites from three matrices in different spiked levels

| Matrix | Spiked level  (mg/kg) | fipronil | | MB46136 | | MB45950 | | MB46513 | |
| --- | --- | --- | --- | --- | --- | --- | --- | --- | --- |
|  |  | Retention Time | Area | Retention Time | Area | Retention Time | Area | Retention Time | Area |
| cottonseed | 0,005ppm-jb-1 | 1.92 | 486.29 | 2.07 | 2006.048 | 2.09 | 1743.334 | 2.01 | 1016.311 |
|  | 0,005ppm-jb-2 | 1.91 | 454.305 | 2.07 | 2097.097 | 2.09 | 1685.51 | 2 | 1025.691 |
|  | 0,005ppm-jb-3 | 1.91 | 490.708 | 2.07 | 2115.983 | 2.09 | 1727.638 | 2 | 1058.701 |
|  | 0,005ppm-tj-1-1 | 1.91 | 468.066 | 2.07 | 1735.298 | 2.09 | 1449.581 | 2 | 959.217 |
|  | 0,005ppm-tj-1-2 | 1.91 | 403.937 | 2.07 | 1644.616 | 2.09 | 1429.649 | 2 | 854.337 |
|  | 0,005ppm-tj-2-1 | 1.91 | 363.161 | 2.07 | 1546.617 | 2.09 | 1370.143 | 2 | 781.74 |
|  | 0,005ppm-tj-2-2 | 1.91 | 350.567 | 2.07 | 1558.697 | 2.09 | 1171.207 | 2 | 853.941 |
|  | 0,005ppm-tj-3-1 | 1.91 | 437.744 | 2.07 | 1775.567 | 2.09 | 1431.95 | 2 | 1000.229 |
|  | 0,005ppm-tj-3-2 | 1.91 | 410.374 | 2.06 | 1855.088 | 2.09 | 1437.051 | 2 | 948.333 |
|  | 0,005ppm-tj-4-1 | 1.91 | 410.816 | 2.06 | 1687.523 | 2.09 | 1337.603 | 2 | 923.577 |
|  | 0,005ppm-tj-4-2 | 1.91 | 403.358 | 2.06 | 1767.521 | 2.08 | 1301.369 | 2 | 951.697 |
|  | 0,005ppm-tj-5-1 | 1.91 | 340.127 | 2.06 | 1716.665 | 2.09 | 1288.433 | 2 | 762.777 |
|  | 0,005ppm-tj-5-2 | 1.91 | 366.036 | 2.06 | 1676.682 | 2.08 | 1284.761 | 2 | 788.006 |
|  | 0,05ppm-jb-1 | 1.91 | 46247.184 | 2.07 | 11916.694 | 2.09 | 7110 | 2 | 5003.212 |
|  | 0,05ppm-jb-2 | 1.91 | 2600.14 | 2.06 | 10608.052 | 2.09 | 6235.413 | 2 | 4221.858 |
|  | 0,05ppm-jb-3 | 1.91 | 3715.553 | 2.06 | 13225.336 | 2.09 | 7992.377 | 2 | 5786.488 |
|  | 0,05ppm-tj-1-1 | 1.91 | 2631.956 | 2.06 | 10700.975 | 2.09 | 6166.329 | 2 | 4258.821 |
|  | 0,05ppm-tj-1-2 | 1.91 | 2600.14 | 2.06 | 10608.052 | 2.09 | 6235.413 | 2 | 4221.858 |
|  | 0,05ppm-tj-2-1 | 1.91 | 2924.609 | 2.06 | 12057.335 | 2.09 | 6867.723 | 2 | 4697.275 |
|  | 0,05ppm-tj-2-2 | 1.91 | 2793.472 | 2.06 | 12157.945 | 2.09 | 6706.227 | 2 | 4384.036 |
|  | 0,05ppm-tj-3-1 | 1.91 | 2197.522 | 2.06 | 9450.866 | 2.09 | 5609.101 | 2 | 3409.821 |
|  | 0,05ppm-tj-3-2 | 1.91 | 2183.055 | 2.06 | 9611.495 | 2.09 | 5733.298 | 2 | 3481.91 |
|  | 0,05ppm-tj-4-1 | 1.91 | 2695.981 | 2.06 | 11747.569 | 2.09 | 6970.457 | 2 | 4527.973 |
|  | 0,05ppm-tj-4-2 | 1.9 | 2511.472 | 2.06 | 11381.252 | 2.09 | 6674.729 | 2 | 4064.379 |
|  | 0,05ppm-tj-5-1 | 1.91 | 2740.65 | 2.06 | 12064.749 | 2.09 | 7089.703 | 2 | 4616.569 |
|  | 0,05ppm-tj-5-2 | 1.91 | 2887.628 | 2.06 | 11739.945 | 2.08 | 6946.676 | 2 | 4631.049 |
|  | 0,5ppm-jb-1 | 1.92 | 58309.547 | 2.07 | 49701.805 | 2.1 | 30125.01 | 2.01 | 50782.332 |
|  | 0,5ppm-tj-2 | 1.92 | 50663.133 | 2.07 | 53090.941 | 2.1 | 27265.16 | 2.01 | 46947.074 |
|  | 0,5ppm-tj-3 | 1.92 | 46686.496 | 2.07 | 55219.793 | 2.1 | 28913.16 | 2.01 | 57024.297 |
|  | 0,5ppm-tj-1-1 | 1.92 | 58483.641 | 2.07 | 51148.195 | 2.1 | 34278.977 | 2.01 | 58742.238 |
|  | 0,5ppm-tj-1-2 | 1.92 | 56598.934 | 2.07 | 48954.457 | 2.1 | 33356.25 | 2.01 | 56248.395 |
|  | 0,5ppm-tj-1-3 | 1.92 | 54427.262 | 2.07 | 47517.77 | 2.1 | 33065.73 | 2.01 | 54782.738 |
|  | 0,5ppm-tj-2-1 | 1.92 | 49512.496 | 2.07 | 43138.602 | 2.1 | 30027.857 | 2.01 | 49733.891 |
|  | 0,5ppm-tj-2-2 | 1.92 | 51036.832 | 2.07 | 44041.477 | 2.1 | 30327.453 | 2.01 | 50150.055 |
|  | 0,5ppm-tj-2-3 | 1.92 | 46878.738 | 2.07 | 41644.973 | 2.1 | 28021.043 | 2.01 | 46851.418 |
|  | 0,5ppm-tj-3-1 | 1.92 | 46755.602 | 2.07 | 41286.18 | 2.1 | 28256.844 | 2.01 | 47024.883 |
|  | 0,5ppm-tj-3-2 | 1.92 | 43473.809 | 2.07 | 39539.93 | 2.09 | 26494.496 | 2.01 | 43132.723 |
|  | 0,5ppm-tj-3-3 | 1.91 | 45557.039 | 2.07 | 40355.867 | 2.09 | 27171.18 | 2.01 | 45264.805 |
|  | 0,5ppm-tj-4-1 | 1.92 | 50663.133 | 2.07 | 43725.379 | 2.1 | 29810.295 | 2.01 | 49708.855 |
|  | 0,5ppm-tj-4-2 | 1.92 | 46686.496 | 2.07 | 42475.438 | 2.1 | 27893.098 | 2 | 46937.645 |
|  | 0,5ppm-tj-4-3 | 1.92 | 49080.043 | 2.07 | 42204.754 | 2.1 | 29238.607 | 2.01 | 46977.332 |
|  | 0,5ppm-tj-5-1 | 1.91 | 41520.082 | 2.07 | 38023.461 | 2.09 | 25857.408 | 2 | 40694.863 |
|  | 0,5ppm-tj-5-2 | 1.91 | 39063.234 | 2.06 | 36995.637 | 2.09 | 25161.449 | 2 | 38280.516 |
|  | 0,5ppm-tj-5-3 | 1.91 | 39131.18 | 2.07 | 36402.801 | 2.09 | 25060.766 | 2.01 | 39175.672 |
| Matrix | Spiked level  (mg/kg) | fipronil | | MB46136 | | MB45950 | | MB46513 | |
| cotton plant |  | Retention Time | Area | Retention Time | Area | Retention Time | Area | Retention Time | Area |
|  | 0,01ppm-jb-1 | 1.91 | 5790.919 | 2.06 | 3680.995 | 2.08 | 2885.237 | 1.99 | 2794.3 |
|  | 0,01ppm-jb-2 | 1.91 | 6205.722 | 2.06 | 4045.714 | 2.09 | 3235.578 | 2 | 3062.936 |
|  | 0,01ppm-jb-3 | 1.91 | 8072.803 | 2.06 | 5132.77 | 2.08 | 4057.624 | 2 | 3678.784 |
|  | 0,01ppm-jb-4 | 1.91 | 8424.539 | 2.06 | 5123.533 | 2.09 | 4244.162 | 2 | 3961.835 |
|  | 0,01ppm-tj-1-1 | 1.91 | 5821.229 | 2.06 | 3874.367 | 2.08 | 3030.198 | 2 | 2955.764 |
|  | 0,01ppm-tj-1-2 | 1.91 | 5972.354 | 2.06 | 3903.912 | 2.08 | 3223.561 | 2 | 3077.003 |
|  | 0,01ppm-tj-2-1 | 1.91 | 6253.958 | 2.06 | 3981.663 | 2.08 | 3309.643 | 2 | 3025.09 |
|  | 0,01ppm-tj-2-2 | 1.91 | 6012.386 | 2.06 | 3864.992 | 2.08 | 3098.572 | 2 | 2831.794 |
|  | 0,01ppm-tj-3-1 | 1.91 | 6722.904 | 2.06 | 4163.655 | 2.08 | 3322.469 | 2 | 3151.293 |
|  | 0,01ppm-tj-3-2 | 1.91 | 6467.937 | 2.06 | 4067.69 | 2.08 | 3447.483 | 2 | 3136.345 |
|  | 0,01ppm-tj-4-1 | 1.91 | 7175.701 | 2.06 | 4303.015 | 2.09 | 3935.328 | 2 | 4059.731 |
|  | 0,01ppm-tj-4-2 | 1.91 | 6798.32 | 2.06 | 4475.232 | 2.08 | 4010.834 | 2 | 4147.564 |
|  | 0,01ppm-tj-5-1 | 1.91 | 7064.308 | 2.06 | 4585.546 | 2.08 | 3803.406 | 2 | 3430.981 |
|  | 0,01ppm-tj-5-2 | 1.91 | 6938.226 | 2.06 | 4415.367 | 2.08 | 3575.44 | 2 | 3359.508 |
|  | 0,05ppm-jb-1 | 1.91 | 19902.58 | 2.06 | 17915.99 | 2.08 | 5530.767 | 2 | 3667.714 |
|  | 0,05ppm-jb-2 | 1.91 | 19567.39 | 2.06 | 18437.84 | 2.08 | 5451.036 | 2 | 3480.974 |
|  | 0,05ppm-jb-3 | 1.91 | 20257.23 | 2.06 | 18455.15 | 2.09 | 5315.224 | 2 | 3330.796 |
|  | 0,05ppm-tj-1-1 | 1.91 | 21418.96 | 2.06 | 19372.25 | 2.09 | 5138.695 | 2 | 3050.882 |
|  | 0,05ppm-tj-1-2 | 1.91 | 21469.82 | 2.06 | 19298.98 | 2.08 | 5239.14 | 2 | 3025.964 |
|  | 0,05ppm-tj-2-1 | 1.91 | 20594.02 | 2.06 | 18893.43 | 2.09 | 5139.102 | 2 | 3080.418 |
|  | 0,05ppm-tj-2-2 | 1.91 | 19709.47 | 2.06 | 18979.25 | 2.08 | 4985.227 | 2 | 3106.868 |
|  | 0,05ppm-tj-3-1 | 1.91 | 19569.94 | 2.06 | 18605.23 | 2.09 | 5007.456 | 2 | 3104.482 |
|  | 0,05ppm-tj-3-2 | 1.91 | 19381.86 | 2.06 | 17738.24 | 2.09 | 5067.46 | 2 | 3140.719 |
|  | 0,05ppm-tj-4-1 | 1.91 | 18907.48 | 2.06 | 17227.72 | 2.08 | 4912.767 | 2 | 3149.422 |
|  | 0,05ppm-tj-4-2 | 1.91 | 18700.71 | 2.06 | 17278.73 | 2.09 | 5058.889 | 2 | 3132.835 |
|  | 0,05ppm-tj-5-1 | 1.91 | 19378.45 | 2.06 | 18176.84 | 2.09 | 4846.768 | 2 | 2832.477 |
|  | 0,05ppm-tj-5-2 | 1.91 | 19477.91 | 2.06 | 17676.94 | 2.08 | 5009.224 | 2 | 2894.852 |
|  | 0,5ppm-jb-1 | 1.91 | 113623.5 | 2.07 | 110350.6 | 2.09 | 29994.04 | 2 | 20324.34 |
|  | 0,5ppm-jb-2 | 1.91 | 119060.6 | 2.07 | 108772.8 | 2.09 | 28493.59 | 2 | 20564.47 |
|  | 0,5ppm-jb-3 | 1.91 | 118450.5 | 2.06 | 111905 | 2.09 | 27498.6 | 2 | 20046.72 |
|  | 0,5ppm-tj-1-1 | 1.91 | 114572.5 | 2.06 | 103361.9 | 2.09 | 25337 | 2 | 18235.27 |
|  | 0,5ppm-tj-1-2 | 1.91 | 113284.3 | 2.07 | 103392.1 | 2.09 | 25512.93 | 2 | 17664.03 |
|  | 0,5ppm-tj-2-1 | 1.91 | 117373.6 | 2.07 | 104851.3 | 2.09 | 28657.85 | 2 | 22372.82 |
|  | 0,5ppm-tj-2-2 | 1.91 | 117484.6 | 2.06 | 102594.6 | 2.09 | 29397.28 | 2 | 22801.13 |
|  | 0,5ppm-tj-3-1 | 1.91 | 115983.4 | 2.06 | 102388.2 | 2.09 | 28061.59 | 2 | 22239.45 |
|  | 0,5ppm-tj-3-2 | 1.91 | 124127.7 | 2.06 | 110513.8 | 2.09 | 29435.42 | 2 | 22953.56 |
|  | 0,5ppm-tj-4-1 | 1.91 | 121644.1 | 2.06 | 108507.4 | 2.09 | 27944.55 | 2 | 21023.62 |
|  | 0,5ppm-tj-4-2 | 1.91 | 119676.1 | 2.06 | 109132 | 2.09 | 28417.14 | 2 | 22272.79 |
|  | 0,5ppm-tj-5-1 | 1.91 | 126662.5 | 2.06 | 115805.1 | 2.09 | 30225.34 | 2 | 22943.56 |
|  | 0,5ppm-tj-5-2 | 1.91 | 123287.7 | 2.06 | 109822.7 | 2.09 | 29597.76 | 2 | 22408.05 |
| Matrix | Spiked level  (mg/kg) | fipronil | | MB46136 | | MB45950 | | MB46513 | |
| soil |  | Retention Time | Area | Retention Time | Area | Retention Time | Area | Retention Time | Area |
|  | 0,005ppm-jb-1 | 1.91 | 6815.504 | 2.06 | 6534.792 | 2.09 | 2000.269 | 2 | 1367.951 |
|  | 0,005ppm-jb-2 | 1.91 | 8306.92 | 2.06 | 6931.122 | 2.09 | 3904.998 | 2 | 1988.599 |
|  | 0,005ppm-jb-3 | 1.91 | 7363.494 | 2.06 | 5740.824 | 2.09 | 3255.47 | 2 | 1437.234 |
|  | 0,005ppm-tj-1-1 | 1.91 | 7776.458 | 2.06 | 6417.31 | 2.09 | 3513.559 | 2 | 1866.925 |
|  | 0,005ppm-tj-1-2 | 1.91 | 7995.455 | 2.06 | 6475.269 | 2.09 | 3581.052 | 2 | 1749.337 |
|  | 0,005ppm-tj-2-1 | 1.91 | 6086.311 | 2.06 | 5307.594 | 2.09 | 2730.227 | 2 | 1192.983 |
|  | 0,005ppm-tj-2-2 | 1.91 | 6107.429 | 2.06 | 5085.759 | 2.09 | 2726.023 | 2 | 1456.06 |
|  | 0,005ppm-tj-3-1 | 1.91 | 6735.208 | 2.06 | 5942.611 | 2.09 | 3388.857 | 2 | 1548.627 |
|  | 0,005ppm-tj-3-2 | 1.91 | 6687.583 | 2.06 | 5882.424 | 2.09 | 3242.434 | 2 | 1701.022 |
|  | 0,005ppm-tj-4-1 | 1.91 | 7294.822 | 2.06 | 5879.121 | 2.09 | 3506.52 | 2 | 1703.87 |
|  | 0,005ppm-tj-4-2 | 1.91 | 7921.66 | 2.06 | 7172.842 | 2.09 | 3651.229 | 2 | 1743.832 |
|  | 0,005ppm-tj-5-1 | 1.91 | 7235.531 | 2.06 | 5496.062 | 2.09 | 2972.832 | 2 | 1752.962 |
|  | 0,005ppm-tj-5-2 | 1.91 | 7950.505 | 2.06 | 6838.754 | 2.09 | 3938.392 | 2 | 2116.659 |
|  | 0,05ppm-jb-1 | 1.91 | 47550.348 | 2.06 | 34017.883 | 2.08 | 23431.145 | 1.99 | 26257.594 |
|  | 0,05ppm-jb-2 | 1.91 | 46030.039 | 2.06 | 29727.545 | 2.08 | 29427.486 | 1.99 | 34742.516 |
|  | 0,05ppm-jb-3 | 1.91 | 47594.66 | 2.06 | 30103.773 | 2.08 | 29724.049 | 2 | 34768.355 |
|  | 0,05ppm-tj-1-1 | 1.91 | 46671.086 | 2.06 | 34744.934 | 2.08 | 25552.938 | 1.99 | 28343.764 |
|  | 0,05ppm-tj-1-2 | 1.91 | 47854.832 | 2.06 | 34619.039 | 2.08 | 29412.262 | 2 | 31149.555 |
|  | 0,05ppm-tj-2-1 | 1.91 | 37313.172 | 2.06 | 24745.514 | 2.08 | 25924.199 | 1.99 | 29957.396 |
|  | 0,05ppm-tj-2-2 | 1.91 | 46472.66 | 2.06 | 32243.252 | 2.08 | 27394.068 | 2 | 30593.332 |
|  | 0,05ppm-tj-3-1 | 1.91 | 46784.582 | 2.06 | 34171.504 | 2.08 | 25766.574 | 2 | 28713.957 |
|  | 0,05ppm-tj-3-2 | 1.91 | 47135.953 | 2.06 | 34028.648 | 2.08 | 26163.393 | 2 | 29747.557 |
|  | 0,05ppm-tj-4-1 | 1.91 | 48609.934 | 2.06 | 33020.543 | 2.08 | 22575.398 | 2 | 26928.34 |
|  | 0,05ppm-tj-4-2 | 1.91 | 47405.977 | 2.06 | 33142.48 | 2.08 | 22262.037 | 1.99 | 26722.422 |
|  | 0,05ppm-tj-5-1 | 1.91 | 46382.52 | 2.06 | 31749.703 | 2.08 | 24688.158 | 2 | 31083.922 |
|  | 0,05ppm-tj-5-2 | 1.91 | 46337.512 | 2.06 | 31468.039 | 2.08 | 23420.486 | 2 | 29815.18 |
|  | 0,5ppm-jb-1 | 1.91 | 151254.6 | 2.07 | 94254.539 | 2.09 | 92162.2 | 2 | 157955.234 |
|  | 0,5ppm-jb-2 | 1.91 | 151802.7 | 2.07 | 87130.273 | 2.09 | 95560.69 | 2 | 148063.859 |
|  | 0,5ppm-jb-3 | 1.92 | 151528.6 | 2.07 | 87542.727 | 2.1 | 93861.45 | 2 | 152657.563 |
|  | 0,5ppm-tj-1-1 | 1.91 | 142888.4 | 2.07 | 91866.078 | 2.09 | 88988.84 | 2 | 124069.594 |
|  | 0,5ppm-tj-1-2 | 1.91 | 133299.5 | 2.07 | 84691.484 | 2.09 | 87047.56 | 2 | 123323.297 |
|  | 0,5ppm-tj-2-1 | 1.92 | 141305.8 | 2.07 | 88159.906 | 2.09 | 86347.3 | 2 | 119420.242 |
|  | 0,5ppm-tj-2-2 | 1.91 | 141296.8 | 2.07 | 91332 | 2.1 | 86997.86 | 2 | 121396.422 |
|  | 0,5ppm-tj-3-1 | 1.91 | 135533.6 | 2.07 | 84838.766 | 2.1 | 90484.6 | 2 | 125829.797 |
|  | 0,5ppm-tj-3-2 | 1.91 | 144089.8 | 2.07 | 87524.063 | 2.09 | 93450.46 | 2 | 130988.523 |
|  | 0,5ppm-tj-4-1 | 1.91 | 138857.3 | 2.07 | 86745.453 | 2.09 | 83415.66 | 2 | 118377.297 |
|  | 0,5ppm-tj-4-2 | 1.91 | 141236.5 | 2.07 | 87315.523 | 2.1 | 83297.88 | 2 | 117450.891 |
|  | 0,5ppm-tj-5-1 | 1.91 | 143603.8 | 2.07 | 89603.828 | 2.09 | 86780.53 | 2 | 122564.375 |
|  | 0,5ppm-tj-5-2 | 1.91 | 133283.8 | 2.07 | 86128.547 | 2.09 | 82554.84 | 2 | 115260.445 |
