# Supplementary material for: Residue analysis and persistence evaluation of fipronil and its metabolites in cotton using high-performance liquid chromatography-tandem mass spectrometry
Source: PLoS One. 2017 Mar 14;12(3):e0173690. doi: 10.1371/journal.pone.0173690 (PMC5349471; doi:10.1371/journal.pone.0173690)
Supplement: S3 Table — (DOCX) [file pone.0173690.s003.docx]

S3 Table The data of dissipation of fipronil and three metabolites in cotton plant and soil at the Shandong and Henan site.

|  | fipronil | | MB46136 | | MB45950 | | MB46513 | |
| --- | --- | --- | --- | --- | --- | --- | --- | --- |
|  | Retention Time | Area | Retention Time | Area | Retention Time | Area | Retention Time | Area |
| 14-sd-soil-dt-28d-1 | 1.91 | 1928.946 | 2.06 | 9637.821 | 2.09 | 163.298 | 2 | 594.401 |
| 14-sd-soil-dt-28d-2 | 1.91 | 1896.26 | 2.06 | 10765.78 | 2.09 | 203.229 | 2 | 503.377 |
| 14-sd-soil-dt-21d-1 | 1.91 | 5474.631 | 2.06 | 8312.28 | 2.09 | 306.988 | 2 | 785.62 |
| 14-sd-soil-dt-21d-2 | 1.91 | 5574.274 | 2.06 | 8773.932 | 2.09 | 315.759 | 2 | 767.718 |
| 14-sd-soil-dt-14d-1 | 1.91 | 6648.127 | 2.06 | 7098.754 | 2.09 | 247.816 | 2 | 716.875 |
| 14-sd-soil-dt-14d-2 | 1.91 | 6106.1 | 2.06 | 7014.39 | 2.09 | 254.036 | 2 | 830.031 |
| 14-sd-soil-dt-7d-1 | 1.91 | 20050.16 | 2.06 | 9445.02 | 2.09 | 522.161 | 2 | 2698.9 |
| 14-sd-soil-dt-7d-2 | 1.91 | 20574.93 | 2.06 | 9750.304 | 2.09 | 545.149 | 2 | 2725.805 |
| 14-sd-soil-dt-4d-1 | 1.91 | 27690.71 | 2.06 | 11932.67 | 2.09 | 725.033 | 2 | 1635.282 |
| 14-sd-soil-dt-4d-2 | 1.91 | 29839.61 | 2.06 | 12226.66 | 2.09 | 786.198 | 2 | 1652.162 |
| 14-sd-soil-dt-2d-1 | 1.91 | 29512.38 | 2.06 | 4609.174 | 2.09 | 405.842 | 2 | 664.294 |
| 14-sd-soil-dt-2d-2 | 1.91 | 28835.61 | 2.06 | 4612.018 | 2.09 | 405.272 | 2 | 610.197 |
| 14-sd-soil-dt-1d-1 | 1.91 | 17093.87 | 2.06 | 2467.865 | 2.09 | 239.952 | 2 | 485.545 |
| 14-sd-soil-dt-1d-2 | 1.91 | 13041.98 | 2.06 | 2396.081 | 2.09 | 270.376 | 2 | 494.045 |
| 14-sd-soil-dt-2h-1 | 1.91 | 30664.68 | 2.06 | 1909.26 | 2.09 | 620.513 | 2 | 260.108 |
| 14-sd-soil-dt-2h-2 | 1.91 | 36301.73 | 2.06 | 1976.426 | 2.09 | 581.999 | 2 | 350.28 |
| 14-hn-soil-dt-28d-1 | 1.92 | 42418.1 | 2.07 | 1483.166 | 2.09 | 508.37 | 2.01 | 7.058 |
| 14-hn-soil-dt-28d-2 | 1.91 | 42096.35 | 2.07 | 1342.287 | 2.09 | 538.046 | 2.01 | 4.456 |
| 14-hn-soil-dt-21d-1 | 1.91 | 35238.94 | 2.07 | 2520.523 | 2.09 | 501.199 | 2 | 5.427 |
| 14-hn-soil-dt-21d-2 | 1.91 | 37711.85 | 2.07 | 2529.018 | 2.09 | 497.101 | 2.01 | 8.09 |
| 14-hn-soil-dt-14d-1 | 1.91 | 29148.27 | 2.06 | 2379.47 | 2.09 | 160.969 | 1.99 | 7.625 |
| 14-hn-soil-dt-14d-2 | 1.92 | 29459.04 | 2.07 | 2928.597 | 2.09 | 411.775 | 2.01 | 7.12 |
| 14-hn-soil-dt-7d-1 | 1.91 | 28016.52 | 2.07 | 5176.337 | 2.09 | 760.764 | 2.01 | 9.779 |
| 14-hn-soil-dt-7d-2 | 1.91 | 27618.67 | 2.07 | 6259.733 | 2.09 | 797.572 | 2.04 | 10.674 |
| 14-hn-soil-dt-4d-1 | 1.91 | 25028.1 | 2.07 | 9745.702 | 2.09 | 870.058 | 2.01 | 15.28 |
| 14-hn-soil-dt-4d-2 | 1.92 | 22697.97 | 2.07 | 8165.867 | 2.09 | 835.694 | 1.99 | 10.406 |
| 14-hn-soil-dt-2d-1 | 1.92 | 18760.17 | 2.07 | 13583.49 | 2.09 | 967.821 | 2 | 5.524 |
| 14-hn-soil-dt-2d-2 | 1.91 | 20437.88 | 2.07 | 13915.89 | 2.09 | 1012.377 | 1.99 | 8.47 |
| 14-hn-soil-dt-1d-1 | 1.91 | 13931.85 | 2.07 | 17897.54 | 2.09 | 477.145 | 2.01 | 16.341 |
| 14-hn-soil-dt-1d-2 | 1.92 | 15414.65 | 2.07 | 17900.14 | 2.09 | 502.059 | 2.01 | 8.399 |
| 14-hn-soil-dt-2h-1 | 1.91 | 3919.743 | 2.07 | 10336.45 | 2.09 | 469.36 | 1.99 | 11.828 |
| 14-hn-soil-dt-2h-2 | 1.92 | 3604.398 | 2.07 | 10043.42 | 2.09 | 436.72 | 2.01 | 1.645 |
| 14-sd- plant-dt-28d-1 | 1.91 | 872.354 | 2.07 | 342.184 | 2.09 | 181.049 | 2 | 159.567 |
| 14-sd-plant-dt-28d-2 | 1.91 | 766.909 | 2.07 | 354.107 | 2.09 | 88.599 | 2 | 76.446 |
| 14-sd-plant-dt-21d-1 | 1.91 | 606.225 | 2.06 | 494.811 | 2.09 | 67.556 | 1.99 | 62.076 |
| 14-sd-plant-dt-21d-2 | 1.91 | 617.32 | 2.06 | 494.189 | 2.09 | 78.407 | 2 | 79.557 |
| 14-sd-plant-dt-14d-1 | 1.91 | 608.24 | 2.06 | 317.063 | 2.09 | 35.155 | 2 | 27.857 |
| 14-sd-plant-dt-14d-2 | 1.91 | 543.272 | 2.06 | 296.775 | 2.1 | 44.111 | 2 | 71.984 |
| 14-sd-plant-dt-7d-1 | 1.91 | 3308.603 | 2.07 | 1884.55 | 2.09 | 85.648 | 2 | 373.899 |
| 14-sd-plant-dt-7d-2 | 1.91 | 3026.576 | 2.06 | 1761.378 | 2.09 | 80.02 | 2 | 438.786 |
| 14-sd-plant-dt-4d-1 | 1.91 | 4608.428 | 2.06 | 4186.036 | 2.09 | 227.739 | 2 | 1022.317 |
| 14-sd-plant-dt-4d-2 | 1.91 | 4681.234 | 2.06 | 4099.006 | 2.09 | 251.604 | 2 | 1185.039 |
| 14-sd- plant -dt-2d-1 | 1.91 | 12075.56 | 2.06 | 4746.278 | 2.09 | 138.305 | 2 | 901.08 |
| 14-sd- plant -dt-2d-2 | 1.91 | 12670.58 | 2.06 | 4768.496 | 2.09 | 166.497 | 2 | 937.962 |
| 14-sd-plant-dt-1d-1 | 1.91 | 14575.54 | 2.06 | 6928.189 | 2.09 | 344.382 | 2 | 1993.835 |
| 14-sd-plant-dt-1d-2 | 1.91 | 14560.23 | 2.07 | 6639.825 | 2.09 | 351.465 | 2 | 2048.292 |
| 14-sd-plant-dt-2h-1 | 1.91 | 19807.77 | 2.07 | 5474.773 | 2.09 | 290.647 | 2 | 798.947 |
| 14-sd-plant-dt-2h-2 | 1.91 | 14563.86 | 2.07 | 6638.319 | 2.09 | 351.312 | 2 | 2048.129 |
| 14-hn-plant-dt-28d-1 | 1.92 | 629.296 | 2.07 | 273.717 | 2.08 | 63.29 | 2 | 63.376 |
| 14-hn-plant-dt-28d-2 | 1.91 | 593.694 | 2.06 | 278.795 | 2.09 | 38.371 | 2 | 63.099 |
| 14-hn-plant-dt-21d-1 | 1.92 | 461.784 | 2.07 | 308.563 | 2.08 | 29.378 | 2 | 30.965 |
| 14-hn-plant-dt-21d-2 | 1.91 | 409.147 | 2.07 | 324.636 | 2.09 | 50.902 | 2 | 68.8 |
| 14-hn-plant-dt-14d-1 | 1.91 | 4529.874 | 2.07 | 256.471 | 2.09 | 44.588 | 2.01 | 38.743 |
| 14-hn-plant-dt-14d-2 | 1.91 | 4194.358 | 2.07 | 260.793 | 2.09 | 51.611 | 2 | 48.993 |
| 14-hn-plant-dt-7d-1 | 1.91 | 6798.506 | 2.07 | 1541.014 | 2.09 | 89.828 | 2 | 331.195 |
| 14-hn-plant-dt-7d-2 | 1.91 | 5836.394 | 2.07 | 1336.824 | 2.09 | 135.743 | 2 | 446.256 |
| 14-hn-plant-dt-4d-1 | 1.91 | 7426.507 | 2.07 | 3584.647 | 2.08 | 176.301 | 2 | 777.664 |
| 14-hn-plant-dt-4d-2 | 1.91 | 7438.289 | 2.07 | 3508.673 | 2.09 | 155.865 | 2 | 806.176 |
| 14-hn- plant -dt-2d-1 | 1.91 | 13315.08 | 2.07 | 3256.646 | 2.09 | 343.638 | 2 | 1749.16 |
| 14-hn- plant -dt-2d-2 | 1.91 | 14049.68 | 2.07 | 3758.561 | 2.09 | 294.006 | 2 | 1211.146 |
| 14-hn-plant-dt-1d-1 | 1.91 | 16969.3 | 2.06 | 6060.574 | 2.09 | 346.567 | 2 | 1759.729 |
| 14-hn-plant-dt-1d-2 | 1.91 | 15613.78 | 2.06 | 5318.857 | 2.09 | 434.399 | 2 | 2590.574 |
| 14-hn-plant-dt-2h-1 | 1.91 | 19766.32 | 2.07 | 4365.724 | 2.09 | 384.026 | 2 | 1012.894 |
| 14-hn-plant-dt-2h-2 | 1.91 | 21371.81 | 2.06 | 4800.364 | 2.09 | 297.634 | 2 | 666.941 |
| 15-SD-SOIL-DT-28D-1 | 1.8 | 10002.41 | 1.96 | 35267.07 | 1.99 | 6630.098 | 1.89 | 407.017 |
| 15-SD-SOIL-DT-28D-2 | 1.8 | 9757.303 | 1.96 | 35115.57 | 1.99 | 6559.009 | 1.89 | 395.577 |
| 15-SD-SOIL-DT-28D-3 | 1.8 | 10001.83 | 1.96 | 35275.34 | 1.99 | 6535.805 | 1.89 | 415.731 |
| 15-SD-SOIL-DT-21D-1 | 1.8 | 74363.07 | 1.96 | 349747.7 | 1.99 | 2423.596 | 1.89 | 38868.77 |
| 15-SD-SOIL-DT-21D-2 | 1.8 | 74496.72 | 1.96 | 357048.8 | 1.99 | 2417.337 | 1.89 | 38654.52 |
| 15-SD-SOIL-DT-21D-3 | 1.8 | 74765.65 | 1.96 | 352189.1 | 1.99 | 2422.732 | 1.89 | 38536.6 |
| 15-SD-SOIL-DT-14D-1 | 1.8 | 95841.24 | 1.96 | 326895.1 | 1.99 | 5185.449 | 1.89 | 30010.03 |
| 15-SD-SOIL-DT-14D-2 | 1.8 | 96493.4 | 1.96 | 336979.9 | 1.99 | 5003.193 | 1.89 | 31040.99 |
| 15-SD-SOIL-DT-14D-3 | 1.8 | 97851.92 | 1.96 | 339186.9 | 1.99 | 5111.223 | 1.89 | 32018.13 |
| 15-SD-SOIL-DT-7D-1 | 1.8 | 149253.5 | 1.96 | 314720.8 | 1.99 | 4730.247 | 1.89 | 34185.98 |
| 15-SD-SOIL-DT-7D-2 | 1.79 | 150903.8 | 1.96 | 306735.1 | 1.99 | 4873.473 | 1.89 | 34421.82 |
| 15-SD-SOIL-DT-7D-3 | 1.8 | 146587 | 1.96 | 303139.4 | 1.99 | 4724.348 | 1.89 | 33765.28 |
| 15-SD-SOIL-DT-4D-1 | 1.8 | 147162 | 1.96 | 249231.5 | 1.99 | 4337.862 | 1.89 | 36829.4 |
| 15-SD-SOIL-DT-4D-2 | 1.8 | 144578.2 | 1.96 | 257766.5 | 1.99 | 4544.05 | 1.89 | 35936.78 |
| 15-SD-SOIL-DT-4D-3 | 1.8 | 146215.6 | 1.96 | 255405.6 | 1.99 | 4453.869 | 1.89 | 35880.84 |
| 15-SD-SOIL-DT-2D-1 | 1.79 | 246137.4 | 1.96 | 295145.7 | 1.99 | 5901.412 | 1.89 | 35086.33 |
| 15-SD-SOIL-DT-2D-2 | 1.8 | 238030 | 1.96 | 284276.9 | 1.99 | 5623.712 | 1.89 | 34239.6 |
| 15-SD-SOIL-DT-2D-3 | 1.8 | 251788.9 | 1.96 | 308580.6 | 1.99 | 5927.283 | 1.89 | 36896.61 |
| 15-SD-SOIL-DT-1D-1 | 1.79 | 256554.7 | 1.96 | 410615.4 | 1.99 | 6794.019 | 1.89 | 65412.4 |
| 15-SD-SOIL-DT-1D-2 | 1.79 | 249695.1 | 1.96 | 407498.6 | 1.99 | 7211.102 | 1.89 | 60379.18 |
| 15-SD-SOIL-DT-1D-3 | 1.8 | 248793.8 | 1.96 | 405167.1 | 1.99 | 6754.25 | 1.89 | 59281.59 |
| 15-SD-SOIL-DT-2H-1 | 1.79 | 295917.2 | 1.96 | 486277.5 | 1.99 | 7399.212 | 1.89 | 63825.38 |
| 15-SD-SOIL-DT-2H-2 | 1.8 | 292598.2 | 1.96 | 483379.1 | 1.99 | 7350.367 | 1.89 | 62046.11 |
| 15-SD-SOIL-DT-2H-3 | 1.79 | 279147.4 | 1.96 | 466850.8 | 1.99 | 7251.52 | 1.89 | 60601.63 |
| 15-HN-SOIL-DT-28D-1 | 1.79 | 218.902 | 1.95 | 1468.316 | 1.98 | 45.708 | 1.87 | 10.239 |
| 15-HN-SOIL-DT-28D-2 | 1.78 | 261.834 | 1.95 | 1591.466 | 1.98 | 45.565 | 1.89 | 12.876 |
| 15-HN-SOIL-DT-28D-3 | 1.79 | 270.79 | 1.95 | 1692.52 | 1.97 | 42.104 | 1.89 | 15.899 |
| 15-HN-SOIL-DT-21D-1 | 1.79 | 3397.935 | 1.95 | 7666.689 | 1.98 | 385.21 | 1.88 | 62.967 |
| 15-HN-SOIL-DT-21D-2 | 1.79 | 3308.005 | 1.95 | 7826.594 | 1.98 | 419.153 | 1.88 | 72.254 |
| 15-HN-SOIL-DT-21D-3 | 1.79 | 3598.465 | 1.95 | 7831.929 | 1.98 | 495.572 | 1.88 | 57.728 |
| 15-HN-SOIL-DT-14D-1 | 1.79 | 7857.136 | 1.95 | 7985.308 | 1.98 | 851.274 | 1.88 | 74.297 |
| 15-HN-SOIL-DT-14D-2 | 1.79 | 7786.118 | 1.95 | 8419.32 | 1.97 | 818.896 | 1.87 | 80.522 |
| 15-HN-SOIL-DT-14D-3 | 1.79 | 7691.497 | 1.95 | 8547.104 | 1.98 | 773.94 | 1.88 | 78.591 |
| 15-HN-SOIL-DT-7D-1 | 1.79 | 1135.097 | 1.95 | 5947.491 | 1.97 | 376.115 | 1.88 | 55.934 |
| 15-HN-SOIL-DT-7D-2 | 1.79 | 1126.463 | 1.95 | 5767.845 | 1.98 | 399.045 | 1.88 | 74.562 |
| 15-HN-SOIL-DT-7D-3 | 1.79 | 1149.782 | 1.95 | 5777.567 | 1.98 | 417.052 | 1.88 | 77.531 |
| 15-HN-SOIL-DT-4D-1 | 1.79 | 9081.569 | 1.95 | 14183.79 | 1.98 | 930.618 | 1.88 | 201.367 |
| 15-HN-SOIL-DT-4D-2 | 1.79 | 9387.726 | 1.95 | 14684.37 | 1.98 | 968.531 | 1.88 | 195.162 |
| 15-HN-SOIL-DT-4D-3 | 1.79 | 9294.601 | 1.95 | 14666.46 | 1.98 | 943.06 | 1.88 | 159.07 |
| 15-HN-SOIL-DT-2D-1 | 1.79 | 582.706 | 1.95 | 7129.81 | 1.98 | 454.602 | 1.88 | 60.762 |
| 15-HN-SOIL-DT-2D-2 | 1.79 | 505.746 | 1.95 | 7026.846 | 1.98 | 434.588 | 1.88 | 65.466 |
| 15-HN-SOIL-DT-2D-3 | 1.79 | 570.423 | 1.95 | 6819.452 | 1.98 | 455.435 | 1.88 | 47.741 |
| 15-HN-SOIL-DT-1D-1 | 1.79 | 1700.058 | 1.95 | 2185.027 | 1.97 | 113.651 | 1.88 | 26.078 |
| 15-HN-SOIL-DT-1D-2 | 1.79 | 1632.738 | 1.95 | 2167.008 | 1.98 | 119.439 | 1.88 | 38.125 |
| 15-HN-SOIL-DT-1D-3 | 1.79 | 1571.597 | 1.95 | 2189.941 | 1.98 | 121.23 | 1.89 | 33.756 |
| 15-HN-SOIL-DT-2H-1 | 1.79 | 1435.452 | 1.95 | 1978.547 | 1.97 | 99.546 | 1.88 | 25.674 |
| 15-HN-SOIL-DT-2H-2 | 1.79 | 1235.45 | 1.95 | 1678.451 | 1.98 | 105.379 | 1.88 | 33.453 |
| 15-HN-SOIL-DT-2H-3 | 1.79 | 1632.32 | 1.95 | 1734.532 | 1.98 | 110.321 | 1.89 | 34.875 |
| 15-SD-PLANT-DT-28D-1 | 1.78 | 9004.515 | 1.94 | 3971.877 | 1.96 | 1294.543 | 1.87 | 1174.611 |
| 15-SD-PLANT-DT-28D-2 | 1.78 | 8905.979 | 1.94 | 3988.579 | 1.96 | 1570.214 | 1.87 | 1218.067 |
| 15-SD-PLANT-DT-28D-3 | 1.78 | 8919.901 | 1.94 | 3944.092 | 1.96 | 1402.715 | 1.87 | 1230.237 |
| 15-SD-PLANT-DT-21D-1 | 1.78 | 55616.88 | 1.94 | 13796.48 | 1.96 | 208.833 | 1.87 | 2969.418 |
| 15-SD-PLANT-DT-21D-2 | 1.78 | 54034.22 | 1.94 | 13224.82 | 2.12 | 32.305 | 1.87 | 2847.705 |
| 15-SD-PLANT-DT-21D-3 | 1.78 | 55518.44 | 1.94 | 13182.49 | 1.96 | 285.734 | 1.87 | 2762.352 |
| 15-SD-PLANT-DT-14D-1 | 1.78 | 88430.73 | 1.94 | 53776.68 | 1.96 | 2018.441 | 1.87 | 14643.99 |
| 15-SD-PLANT-DT-14D-2 | 1.78 | 87148.57 | 1.94 | 51713.78 | 1.96 | 2054.854 | 1.87 | 14579.99 |
| 15-SD-PLANT-DT-14D-3 | 1.78 | 86989.73 | 1.94 | 51793.27 | 1.96 | 2075.114 | 1.87 | 14402.44 |
| 15-SD-PLANT-DT-7D-1 | 1.78 | 38061.84 | 1.94 | 27845.02 | 1.96 | 847.343 | 1.87 | 7415.324 |
| 15-SD-PLANT-DT-7D-2 | 1.78 | 37037.02 | 1.94 | 27656.67 | 1.96 | 860.793 | 1.87 | 7310.109 |
| 15-SD-PLANT-DT-7D-3 | 1.78 | 37064.1 | 1.94 | 27571.96 | 1.96 | 996.906 | 1.87 | 7215.331 |
| 15-SD-PLANT-DT-4D-1 | 1.78 | 184998.7 | 1.94 | 93405.32 | 1.96 | 4333.592 | 1.87 | 24459.16 |
| 15-SD-PLANT-DT-4D-2 | 1.78 | 188246.1 | 1.94 | 93649.88 | 1.96 | 4615.295 | 1.87 | 26153.84 |
| 15-SD-PLANT-DT-4D-3 | 1.78 | 185700.6 | 1.94 | 93797.98 | 1.96 | 4764.75 | 1.87 | 27393.3 |
| 15-SD-PLANT-DT-2D-1 | 1.78 | 270522.1 | 1.93 | 148207.5 | 1.96 | 6348.689 | 1.87 | 38252.41 |
| 15-SD-PLANT-DT-2D-2 | 1.78 | 265560.9 | 1.94 | 147111.5 | 1.97 | 6636.292 | 1.87 | 38506.29 |
| 15-SD-PLANT-DT-2D-3 | 1.78 | 268321.6 | 1.94 | 149348.7 | 1.96 | 6520.26 | 1.87 | 39788.03 |
| 15-SD-PLANT-DT-1D-1 | 1.78 | 301120.8 | 1.94 | 154003.9 | 1.96 | 6712.75 | 1.87 | 38240.63 |
| 15-SD-PLANT-DT-1D-2 | 1.78 | 306208.9 | 1.93 | 157962.8 | 1.96 | 7153.346 | 1.87 | 40744.02 |
| 15-SD-PLANT-DT-1D-3 | 1.78 | 298448.7 | 1.93 | 150116.7 | 1.96 | 7097.611 | 1.87 | 40573.35 |
| 15-SD-PLANT-DT-2H-1 | 1.78 | 324436.7 | 1.93 | 178782.3 | 1.96 | 7520.264 | 1.87 | 39290.02 |
| 15-SD-PLANT-DT-2H-2 | 1.78 | 317914 | 1.94 | 172484.5 | 1.96 | 7606 | 1.87 | 38345.74 |
| 15-SD-PLANT-DT-2H-3 | 1.78 | 319903.8 | 1.94 | 175477.8 | 1.96 | 7602.096 | 1.87 | 39359.1 |
| 15-HN-PLANT-DT-28D-1 | 1.78 | 5940.999 | 1.94 | 3465.925 | 1.96 | 94.611 | 1.87 | 453.807 |
| 15-HN-PLANT-DT-28D-2 | 1.78 | 5788.023 | 1.94 | 3451.251 | 1.97 | 79.455 | 1.87 | 440.321 |
| 15-HN-PLANT-DT-28D-3 | 1.78 | 5239.959 | 1.94 | 2801.357 | 1.96 | 266.805 | 1.87 | 476.407 |
| 15-HN-PLANT-DT-21D-1 | 1.78 | 1087.879 | 1.93 | 1197.531 | 2.14 | 25.686 | 1.87 | 33.377 |
| 15-HN-PLANT-DT-21D-2 | 1.78 | 1080.468 | 1.94 | 1140.133 | 2.14 | 17.239 | 1.87 | 28.745 |
| 15-HN-PLANT-DT-21D-3 | 1.78 | 1068.844 | 1.94 | 1074.399 | 2.11 | 12.655 | 1.87 | 42.849 |
| 15-HN-PLANT-DT-14D-1 | 1.78 | 11177.17 | 1.94 | 11086.76 | 1.96 | 97.619 | 1.87 | 112.464 |
| 15-HN-PLANT-DT-14D-2 | 1.78 | 11109.7 | 1.93 | 11360.86 | 1.96 | 112.694 | 1.86 | 103.226 |
| 15-HN-PLANT-DT-14D-3 | 1.78 | 10942.18 | 1.94 | 11194.69 | 1.96 | 99.734 | 1.87 | 121.17 |
| 15-HN-PLANT-DT-7D-1 | 1.78 | 43287.32 | 1.94 | 23453.23 | 1.96 | 2115.171 | 1.87 | 958.352 |
| 15-HN-PLANT-DT-7D-2 | 1.78 | 56234.2 | 1.94 | 23987.12 | 1.97 | 2340.406 | 1.87 | 960.438 |
| 15-HN-PLANT-DT-7D-3 | 1.78 | 40383.43 | 1.93 | 22124.98 | 1.96 | 2258.792 | 1.87 | 1023.986 |
| 15-HN-PLANT-DT-4D-1 | 1.78 | 54236.37 | 1.94 | 39801.24 | 1.96 | 3359.076 | 1.87 | 2022.794 |
| 15-HN-PLANT-DT-4D-2 | 1.78 | 52570.85 | 1.94 | 38586.95 | 1.96 | 3319.485 | 1.87 | 1998.963 |
| 15-HN-PLANT-DT-4D-3 | 1.78 | 51668.3 | 1.94 | 38629.76 | 1.97 | 3364.597 | 1.87 | 1995.496 |
| 15-HN-PLANT-DT-2D-1 | 1.78 | 85660.06 | 1.94 | 51845.66 | 1.96 | 1226.375 | 1.87 | 568.278 |
| 15-HN-PLANT-DT-2D-2 | 1.78 | 86595.91 | 1.94 | 52706.01 | 1.96 | 1240.294 | 1.87 | 543.618 |
| 15-HN-PLANT-DT-2D-3 | 1.78 | 85507.93 | 1.94 | 52653.92 | 1.96 | 1269.493 | 1.87 | 607.039 |
| 15-HN-PLANT-DT-1D-1 | 1.78 | 131461.1 | 1.94 | 75272.63 | 1.96 | 649.798 | 1.87 | 548.523 |
| 15-HN-PLANT-DT-1D-2 | 1.78 | 134404.4 | 1.94 | 76410.68 | 1.96 | 676.553 | 1.87 | 474.098 |
| 15-HN-PLANT-DT-1D-3 | 1.78 | 133560.2 | 1.94 | 77488.93 | 1.96 | 619.789 | 1.87 | 484.926 |
| 15-HN-PLANT-DT-2H-1 | 1.78 | 223032.1 | 1.94 | 93295.06 | 1.96 | 654.18 | 1.87 | 345.12 |
| 15-HN-PLANT-DT-2H-2 | 1.78 | 220552.3 | 1.94 | 93312.23 | 1.96 | 643.32 | 1.87 | 356.67 |
| 15-HN-PLANT-DT-2H-3 | 1.78 | 214529.2 | 1.94 | 90785.77 | 1.96 | 645.12 | 1.87 | 378.23 |
