# Supplementary material for: Residue analysis and persistence evaluation of fipronil and its metabolites in cotton using high-performance liquid chromatography-tandem mass spectrometry
Source: PLoS One. 2017 Mar 14;12(3):e0173690. doi: 10.1371/journal.pone.0173690 (PMC5349471; doi:10.1371/journal.pone.0173690)
Supplement: S4 Table — (DOCX) [file pone.0173690.s004.docx]

S4 Table The data of residue residues of fipronil and its metabolites in cottonseed samples.

| Sample | Site | fipronil | | MB46136 | | MB45950 | | MB46513 | |
| --- | --- | --- | --- | --- | --- | --- | --- | --- | --- |
|  |  | Retention Time | Area | Retention Time | Area | Retention Time | Area | Retention Time | Area |
| Sample 1 | Weifang | 1.82 | 76.602 | 1.97 | 193.77 |  | ND | 1.91 | 17.326 |
| Sample 2 | Weifang | 1.78 | 99.796 | 1.95 | 58.325 | 1.96 | 9.589 | 1.89 | 2.545 |
| Sample 3 | Weifang | 1.79 | 59.882 | 1.94 | 48.001 | 1.97 | 0.98 | 1.92 | 2.247 |
| Sample 4 | Weifang | 1.79 | 125.818 | 1.95 | 61.36 | - | ND | 1.86 | 10.257 |
| Sample 5 | Weifang | 1.78 | 17.449 | 1.93 | 0.596 | - | ND | 1.84 | 10.109 |
| Sample 6 | Weifang | 1.79 | 167.94 | 1.95 | 46.521 | 1.96 | 29.745 | 1.87 | 1.023 |
| Sample 7 | Weifang | 1.78 | 108.234 | 1.95 | 34.267 | 1.95 | 3.56 | 1.87 | 10.751 |
| Sample 8 | Weifang | 1.79 | 548.256 | 1.94 | 190.765 | 1.95 | 26.378 | 1.88 | 109.83 |
| Sample 9 | Qingdao | 1.79 | 216.927 | 1.94 | 85.971 | - | ND | 1.88 | 18.024 |
| Sample 10 | Qingdao | 1.78 | 134.193 | 1.94 | 13.817 | - | ND | 1.87 | 3.28 |
| Sample 11 | Qingdao | 1.78 | 125.574 | 1.93 | 46.714 | - | ND | 1.91 | 21.944 |
| Sample 12 | Qingdao | 1.78 | 93.656 | 1.94 | 50.947 | 1.92 | 2.826 | - | ND |
| Sample 13 | Qingdao | 1.78 | 111.771 | 1.94 | 67.873 | - | ND | 1.87 | 8.877 |
| Sample 14 | Qingdao | 1.78 | 89.166 | 1.94 | 25.192 | - | ND | - | ND |
| Sample 15 | Qingdao | 1.78 | 175.024 | 1.93 | 36.829 | 1.99 | 3.598 | 1.9 | 9.378 |
| Sample 16 | Qingdao | 1.77 | 28.355 | 1.94 | 25.239 | 2.01 | 7.705 | 1.94 | 6.012 |
| Sample 17 | Dongying | 1.77 | 71.008 | 1.94 | 9.034 | 1.98 | 0.423 | 1.85 | 2.226 |
| Sample 18 | Dongying | 1.78 | 157.82 | 1.93 | 14.677 | 1.96 | 19.436 | - | ND |
| Sample 19 | Dongying | 1.78 | 110.701 | 1.93 | 20.401 | 1.9 | 4.945 | - | ND |
| Sample 20 | Dongying | 1.78 | 99.343 | 1.94 | 36.496 | - | ND | - | ND |
| Sample 21 | Dongying | 1.78 | 137.377 | 1.93 | 41.917 | 1.93 | 7.754 | - | ND |
| Sample 22 | Dongying | 1.79 | 74.972 | 1.93 | 17.217 | 1.91 | 1.526 | 1.86 | 3.438 |
| Sample 23 | Dongying | 1.78 | 109.555 | 1.92 | 14.734 | - | ND | 1.86 | 13.993 |
| Sample 24 | Dongying | 1.78 | 44.76 | 1.93 | 35.466 | - | ND | - | ND |
| Sample 25 | Heze | 1.78 | 88.972 | 1.94 | 43.936 | 1.96 | 0.83 | 1.91 | ND |
| Sample 26 | Heze | 1.78 | 159.187 | 1.93 | 17.95 | - | ND | - | ND |
| Sample 27 | Heze | 1.78 | 39.252 | 1.94 | 36.833 | 2.02 | 5.916 | 1.8 | 2.489 |
| Sample 28 | Heze | 1.78 | 45.166 | 1.94 | 2.65 | - | ND | - | ND |
| Sample 29 | Heze | 1.78 | 45.688 | 1.94 | 2.79 | - | ND | - | ND |
| Sample 30 | Heze | 1.78 | 430.169 | 1.94 | 71.205 | 1.99 | 0.254 | 1.86 | 27.549 |
| Sample 31 | Heze | 1.78 | 120.64 | 1.94 | 16.701 | 1.9 | 2.331 | - | ND |
| Sample 32 | Heze | 1.78 | 105.367 | 1.94 | 9.36 | 1.91 | 11.067 | 1.86 | 0.916 |
| Sample 33 | Jining | 1.78 | 122.177 | 1.94 | 44.533 | - | ND | 1.88 | 20.336 |
| Sample 34 | Jining | 1.78 | 131.171 | 1.94 | 50.237 | 2.01 | 2.376 | 1.88 | 14.292 |
| Sample 35 | Jining | 1.78 | 81.382 | 1.92 | 14.968 | - | ND | 1.81 | 5.09 |
| Sample 36 | Jining | 1.77 | 139.336 | 1.92 | 14.655 | 1.96 | 0.793 | - | ND |
| Sample 37 | Jining | 1.77 | 46.098 | 1.93 | 33.866 | 1.95 | 0.238 | - | ND |
| Sample 38 | Jining | 1.77 | 78.83 | 1.93 | 8.794 | - | ND | - | ND |
| Sample 39 | Jining | 1.78 | 56.593 | 1.92 | 7.061 | 1.94 | 0.695 | - | ND |
| Sample 40 | Jining | 1.77 | 424.291 | 1.93 | 104.621 | - | ND | 1.87 | 26.664 |
